# Prescribing tailored home exercise program to older adults in the community using a tailored self-modeled video: A pre-post study

**DOI:** 10.3389/fpubh.2022.974512

**Published:** 2022-12-22

**Authors:** Sharmila Vaz, Jo-Aine Hang, Jim Codde, David Bruce, Katrina Spilsbury, Anne-Marie Hill

**Affiliations:** ^1^School of Allied Health, WA Centre for Health and Ageing, The University of Western Australia, Perth, WA, Australia; ^2^Institute for Health Research, The University of Notre Dame, Fremantle, WA, Australia; ^3^Medical School, The University of Western Australia, Perth, WA, Australia

**Keywords:** aging, audiovisual demonstration, exercise therapy, frail elderly, functional decline, patient discharge, rehabilitation

## Abstract

**Background:**

Community rehabilitation for older people after hospital discharge is necessary to regain functional ability and independence. However, poor adherence to exercise programs continues to hinder achieving positive health outcomes in older people. This study aimed to evaluate the effectiveness of prescribing a tailored video self-modeled DVD-HEP for 6 weeks, on functional mobility, physical activity, exercise self-efficacy, and health-related quality of life, in a sample of frail older adults.

**Materials and methods:**

A pre- and post-test intervention study design was conducted, with each participant acting as their own control. A convergent, parallel, mixed-methods approach involving quantitative, and qualitative data collection was used. Participants received an individualized assessment at baseline and subsequently were provided with a 30-min tailored 6-week self-modeled DVD-HEP that showed the physiotherapist instructing the participant. The physiotherapist phoned participants fortnightly to encourage engagement in the program and explore responses to it. Outcomes evaluated included functional mobility, balance, gait speed, and exercise self-efficacy.

**Results:**

Participants (*n* = 15) showed clinically meaningful improvements at follow-up compared to baseline in functional mobility (TUG_MCID_ = 3.4–3.5 s, 3-MWT_MCID_ = 0.1–0.2 m/s) and gait speed (3-MWT_MCID_ = 0.1–0.2 m/s). There were also significant improvements in balance and self-efficacy for exercise and a 2.5- and a 1.3-fold increase in moderate and light physical activity participation at follow-up compared to baseline. The deductive themes were: (i) Enjoyment, self-efficacy, and wellbeing; (ii) Achieving life goals; (iii) Background music as a motivator to adherence; and (iv) Enhanced motor performance and learning: Task goal mastery, multimodal feedback, autonomy to self-regulate learning. The new inductive theme was (v) Preference for in-person support for exercise.

**Conclusion:**

Future studies are warranted to compare a tailored self-modeled video HEP to face-to-face programs and other digital health modalities to evaluate older adults' adherence levels and functional improvement.

## 1. Background

Aging is not only associated with an increased risk of chronic disease and functional and cognitive decline (1) but also significant health service utilization (2). For example, in 2019–2020, hospitalization rates in Australians aged 65 years and over per 1,000 ranged from 981 to 1,469 per 1,000 person-years, compared with 84 to 598 per 1,000 person-years in persons aged <65 years (3). Between 30 and 60% of older patients experience functional decline after hospitalization, resulting in reduced health-related quality of life and autonomy (4, 5).

Community rehabilitation of older people post-discharge is one of the fundamental approaches to reversing functional decline and improving independence. Exercise and physical activity are established strategies for healthy aging (6, 7). Exercise improves functional mobility, including strength and balance, and positively impacts participation in activities of daily living (1, 8–10).

Self-directed home exercise programs (HEP) are often prescribed to maximize recovery and ensure the maintenance of therapeutic gains produced during supervised treatment (11). The findings of a large randomized controlled trial in older people discharged from hospital rehabilitation wards suggest that tailored health professional education alone is insufficient to drive behavior change, with participants reporting several social and health care barriers to functional recovery and engagement in exercise (12, 13). Furthermore, poor adherence to a HEP continues to significantly hinder the achievement of favorable health outcomes in older people (14). Systematic reviews suggest that the proportion of older adults completing group exercise programs ranged from 65 to 86%, the proportion of sessions attended ranged from 58 to 77%, and the average number of HEP sessions completed ranged from 1.5 to 3 times/week (15, 16). Older people need responsive programs in their local community with wrap-around support (12, 13). Also, for older people to be engaged in exercise, the program needs to be fun and meaningful (17), with exercises tailored to target the individual's needs, preferences, interests, and learning styles (18, 19).

There is emerging evidence supporting the role of digital health technologies as safe and effective models of exercise delivery in older people with multimorbidity (20). Technologies like telephone and videoconferencing are expensive and time-consuming as they need synchronous contact with a health professional (21). While smartphones or online applications can reduce healthcare costs by automating processes (prescription and monitoring progress), older people may face limited uptake due to unfamiliarity and the absence of human engagement (22). A mixed-method study exploring older people's preferences about desired modes to receive HEP found that most selected the video delivery over the combined video and paper formats and the paper-only form (23). The video HEP was chosen for its visual appeal and easy-to-follow instructions. Typically, generic video modeling—with peers or others was used. The combination of video and paper enabled a more comprehensive understanding of HEP—with the video footage showing participants *how* to practice an exercise, and written instructions helped them understand the *why* and remember the *how* (23).

Video self-modeling is a form of observational learning in which individuals observe themselves performing a targeted behavior successfully on video and subsequently imitate the behavior (24). Video self-modeling allows individuals to view themselves as successful, acting appropriately, or performing new tasks. Video self-modeling has been successfully used in professions outside of healthcare. For example, within the sporting context, video self-modeling has been shown to improve tactical skill and knowledge more quickly than coaching (25, 26) and enable deeper self-reflection and learning in structured clinical settings (27). There is, however, limited evidence to support the effect of a tailored self-modeled video HEP for older adults (28, 29).

We previously conducted a small feasibility study using a convenience sample of four older people and provided a 5-week tailored video self-modeled DVD-HEP (30). Participants showed a high adherence to the DVD-HEP over the 5-week intervention. Adherence was enhanced by physical improvement, positive self-reflection about the DVD-HEP, and increased self-efficacy (30). The study concluded that tailored self-modeling videos might be feasible to promote adherence to HEP in community-dwelling older patients post-hip fracture (30). We built on this prior work (30) to assess the impact of a tailored self-modeled DVD-HEP in improving functional outcomes in a larger sample of frail older adults relative to their baseline scores. The current study aimed to evaluate the effectiveness of prescribing a tailored self-modeled DVD-HEP for 6-weeks on functional mobility, physical activity, exercise self-efficacy, and health-related quality of life. The secondary aim of the study was to evaluate adherence to the tailored self-modeled DVD-HEP throughout the 6-week intervention.

## 2. Trial registration

The study was registered with the Australian New Zealand Clinical Trials Registry with the registration number: Trial Id: ACTRN12616000946415.

## 3. Materials and methods

### 3.1. Design

A pre- and post-test intervention study was conducted, with each participant acting as their own control. A convergent, parallel, mixed-methods approach was used, involving quantitative and qualitative data collection (31).

### 3.2. Participants and setting

A convenience sample was identified from older adults who sustained a fracture necessitating hospital admission or who experienced functional decline following hospital admission, illness, or a scheduled medical check-up at an outpatient clinic. Recruitment took place from an outpatient aged-care rehabilitation clinic attached to a secondary hospital in metropolitan Perth, Western Australia (WA). All potential participants visited their geriatrician for a scheduled medical check-up or follow-up after hospital discharge and had completed either an inpatient or outpatient rehabilitation program, including follow-up therapy.

*Eligibility criteria for inclusion included*: (i) aged between 60 and 95 years; (ii) able to speak and understand English; (iii) cognitive ability to engage in a self-directed program [eligible if mini-mental state examination score (MMSE) > 23/30] (32); (iv) not currently participating or completing an exercise program; (v) history of prior completion of a rehabilitation program *via* an outpatient rehabilitation clinic or private therapy clinic; (vi) assessed by the hospital geriatrician to be medically stable; and (vii) below their pre-morbid functional level of activity.

*Participants were excluded if they demonstrated*: (i) sensory deficits (audio or visual) that could not be overcome with correction (e.g., glasses or hearing aids); or (ii) a known medical diagnosis that either predisposed them to a high risk of falls or precluded them from safely and independently following a HEP. These included patients diagnosed with Parkinson's disease, a recent history of stroke, or postural hypotension. On reviewing a participant's medical records, if there were queries about their safety in using a HEP, the research team consulted with the hospital geriatrician to decide eligibility.

### 3.3. Intervention

The intervention comprised a 6-week structured HEP, delivered *via* a 30-min video recording on a DVD—henceforth referred to as a DVD-HEP. A JVC camcorder (model no: GZ-HM670BAA) was used to record the video of each customized training routine provided by the therapist, which consisted of a combination of widescreen and close shots of each participant that emphasized key points. The recording was undertaken by a research assistant, who received training from the university's media team to ensure quality control. It took ~1–1.5 h to record each HEP and ~3 h of production and editing time to convert it to DVD format. Details about the software and editing can be found in Supplementary material 1.

Each DVD-HEP included a tailored introduction to the exercise program provided by the same physiotherapist, followed by video footage of the participant performing the tailored exercises. The therapist also provided personalized feedback on how the participant executed each exercise. The physiotherapist also appeared in the video and provided the participant with brief, timely, and explicit guidance to facilitate the correct execution of techniques. Feedback included instructions on improving movement accuracy, suggestions on compensatory movements to avoid or contraindications to consider, reminders, encouragement, and visual cues. Although intervention intensity varied across participants, given that 80% of our sample had sustained a fall in the past year, evidence-based intervention components to reduce falls were included in the videos—focusing on lower extremities strength, balance, postural control, and walking (33).

Factors considered while designing the DVD-HEP to ensure fidelity and feasibility are attached in Supplementary material 1 (30). Exercises were designed to be completed with materials readily available at home, including a chair, wall, or bench for handholds. In addition, in line with neuroscience evidence on the benefits of background music for brain plasticity and as a motivator for exercise (34, 35), participants were asked to provide the physiotherapist with their favorite motivational instrumental music track, which was played in the background. The volume of the background music was adjusted to ensure that it did not overpower or clash with verbal cues. Instrumental music was selected to minimize participant distraction or unintended attention to voices and sung lyrics.

### 3.4. Data collection

#### 3.4.1. Demographics

Demographic data were collected for all participants, including age, gender, the highest level of education, living arrangement, and environment, the number of falls in the last 12 months and whether they sustained a fracture and type of fracture sustained, BMI level and the use of walking aid at home.

#### 3.4.2. Outcomes

##### 3.4.2.1. Primary outcomes

Functional mobility was assessed using Timed Up and Go (TUG) (36), gait speed was assessed using the 3-Meter Walking Test (3-MWT) (37), and balance was measured using the Step Test (38). These outcome measures are validated tools for evaluating community-dwelling older adults' physical function (39, 40).

##### 3.4.2.2. Physical activity

Engagement in physical activity was measured using the Community Healthy Activities Model Program for Seniors (CHAMPS) (41). The measure is suitable for older Australian adults if adequate assistance is provided during administration (42).

##### 3.4.2.3. Exercise self-efficacy

Self-perceived exercise ability and motivation were measured using the Outcome Expectancy for Exercise-2 test (OEE-2) (43), and self-efficacy for exercise was measured using an adapted version of the Self-Efficacy for Exercise scale (SEE) (44, 45). The 13-item OEE-2 was used to rate adults' responses against statements (9-positive items, POEE and 4-negative, NOEE) about the benefits of exercising, using a 5-point Likert scale (0 = strongly agree and 5 = strongly disagree) (46). The SEE scale used in this study rated older adults' confidence about exercise barriers (using an 11-item scale ranging from 0 = not confident to 10 = very confident, with a total score of 110). A sample item includes, “Would you exercise if you felt tired during or after?”

#### 3.4.3. Secondary outcomes

##### 3.4.3.1. Health-related quality of life

Participant perceived health-related Quality of Life (HRQoL) was measured using European Quality of Life-5 Dimensions (EQ-5D-5L) (47).

##### 3.4.3.2. Exercise adherence

Exercise adherence was measured using a daily exercise diary provided at baseline. Participants were asked to complete the log each time they completed an exercise session and, every week, record any reflections about their exercises in the diary.

##### 3.4.3.3. Qualitative

Qualitative feedback from participants about their perceptions and beliefs about completing a HEP using the DVD was explored by reviewing their exercise diaries, fortnightly (3 in total) semi-structured telephone interviews conducted by the physiotherapist and final comments from participants noted down verbatim by the research assistant at the final assessment. Please refer to Supplementary material 2 for an overview of the semi-structured interview schedule.

### 3.5. Procedure

A face-to-face detailed physiotherapy assessment occurred at baseline. Participants' personal and functional goals and expectations were discussed during this session. The physiotherapist demonstrated each exercise included in the HEP and requested the participant to practice it a few times—until the participant could independently execute each exercise using the correct techniques. At the end of the baseline session, participants were given a tailored paper-based copy of the suggested HEP until they received the DVD-HEP copy in the post. Participants were also given an exercise diary to document their HEP participation, thoughts, and feelings. They were advised to practice 30-min of the HEP at least three times a week over six consecutive weeks (a total of 1.5 h a week). Post-test data collection was conducted by the same physiotherapist and occurred 7-weeks after the baseline session to accommodate a week for DVD-HEP production and delivery.

Over the course of 6-weeks, each participant received three phone calls from the therapist to encourage adherence, discuss progress, and provide technical and clinical support. A semi-structured protocol was followed to minimize subjectivity and safeguard data collection fidelity (30). The topic guide followed a framework of participants' use of the DVD, their feelings about the DVD-HEP, and their response to it (Refer to Supplementary material 2: Interview guide). The phone interview allowed participants a convenient and confidential way to discuss their experiences openly and honestly (48).

Each phone call lasted between 20 and 30 min. The first phone call occurred a week after the initial baseline session and coincided with the check-up that the DVD had arrived and could be used by participants. The second and third calls were 2- and 4 weeks post-DVD arrival, respectively. During the last phone call, the physiotherapist arranged the post-test assessment and reminded participants to bring their exercise diaries to the post-test appointment. The therapist made detailed notes from the phone calls, reflected and summarized them immediately afterward, and then performed member checking with participants at the last appointment.

### 3.6. Analysis

Quantitative data were analyzed using SPSS Version 25 for Windows (49). Participants' demographic profile was summarized using descriptive statistics (Table 1). Primary outcome measures of functional mobility (TUG) (36), gait speed (3-MWT) (37), and balance (Step Test) (38) were recorded as raw scores. CHAMPS activity data levels were categorized into four metabolic equivalents of task (MET) levels as either: very light < 2 METS, light > 2 but < 3 METS, moderate > 3 but <6 METS, or vigorous > 6 METS (41). Each completed activity's mean hours per week were recorded and cross-referenced against its corresponding MET level (41). Mean scores were computed to represent self-efficacy and expected outcome scores (SEE) (45, 46) and OEE scores (44). Additionally, positive and negative OEE-2 scale items were computed to represent respective expectations for exercise (46). Quality of life (EQ-5D-5L) was treated using the Dolan method, which allows a single score to be reliably generated for the categorical items that reflect the overall HRQoL and the visual analog scale (VAS) score (47).

**Table 1:** Demographic characteristics of participants^*^.

**Age (mean, SD)**	**80.4 (7.2)**
**Sex (** ***n*** **, %)**	
Female	12 (80)
Male	4 (20)
**Living arrangements (** ***n*** **, %)**	
Alone	10 (63)
With partner	4 (25)
With family members	2 (13)
**Education (** ***n*** **, %)**	
Up to year 10	4 (25)
Completed year 12	6 (38)
Apprenticeship or diploma	4 (25)
University degree	2 (12)
**Number of falls in the last 12 months (** ***n*** **, %)**	
None	3 (19)
One	7 (44)
More than one	6 (38)
**Diagnosis (** ***n*** **, %)**	
Functional decline in last 12 months	7 (56)
Fracture, hip	7 (31)
Fracture, other	2 (13)
**Duration since the last hospital discharge (** ***n*** **, %)**	
<3 months	2 (13)
>3 months	9 (56)
Functional decline identified at the out-patient clinic	5 (31)
**Used a mobility aid (** ***n*** **, %)**	
No	10 (63)
Walking stick	3 (19)
Elbow crutch	1 (6)
Four-wheel walker	2 (13)
**BMI (** ***n*** **, %)**	
Healthy weight	8 (50)
Overweight	3 (19)
Obese	5 (31)

* Total percentages may not add due to rounding.

Given our sample size (*n* = 15), we used the Shapiro-Wilk test to assess normality. Given that all outcomes except the CHAMPS were normally distributed, the differences between baseline and post-intervention performance measures were analyzed using a paired-sample *t-*test. Due to the skewed distribution of CHAMPS scores, the Wilcoxon Signed-Rank test was used to assess change in CHAMPS scores. Additionally, changes in the four CHAMPS activity levels over time were determined using mixed models assuming a negative binomial distribution and treating time (intervention) as a fixed effect and participant and activity levels as random effects. Interaction terms between the time points and activity levels were also included in the model.

Qualitative data were analyzed using inductive and deductive thematic approaches (50). The deductive approach was modeled on the framework used in our earlier feasibility study (30). This framework had been identified as wellness, life goals, and positive impact, with a central theme of the DVD format providing self-efficacy and physical improvements, which promoted adherence to the exercise (30). Participants' anonymized diaries, phone call data, and research assistant notes were independently read by two researchers (JAH, AMH) several times to understand the data. Subsequently, each researcher independently organized codes under the main themes using a stepwise categorization process. They later discussed codes and themes until a consensus was reached (50). An inductive approach was used to analyze unanticipated themes in the data—this involved independent open coding and categorization by the same researchers (JAH, AMH). Finally, a third researcher (JC) was invited to review each reviewer's final codes and themes, followed by discussions by all reviewers until a consensus was reached. An audit trail was maintained to connect the sources (50). Representative exemplary quotes are presented in the results (51).

### 3.7. Ethical considerations

Ethics clearance for the study was obtained from the University of Notre Dame Human Research Ethics Committee (HREC) (Reference number: 015146F) and the South Metropolitan Health Service (SMHS) HREC (Reference Number: 15–190). All participants provided written informed consent to be included in the study.

## 4. Results

Recruitment took place between September 2016 and October 2017. The physiotherapist assessed twenty-five potential participants for eligibility over the phone; 20 met the criteria for inclusion, and four were lost to follow-up after enrolment. Of the 16 participants who completed baseline assessments, one could not attend follow-up assessments, and another did not provide physical activity data. Participants' demographic profile is presented in Table 1. Participants' average age was 80.4 years (Standard deviation, *SD* = 7.2 years). Most participants (80%) were female, and two-thirds (*n* = 10) reported living alone in the community. Eleven participants (69%) had a history of hospitalization the year before the study commenced. Only two of the eleven (19%) reported being discharged from the hospital within 3 months of recruitment. Just under 40% (*n* = 6) used a mobility aid at baseline.

### 4.1. *Post-hoc* sample size justification

Due to hospital changes within the local area health service, the original sample size was reduced to what was expected. Hence a *post-hoc* power calculation was conducted. Our sample size of *n* = 14 in a paired means study design assuming a mean baseline TUG of 17.6 and a standard deviation of the change score of 4.02 had 90% power at alpha 0.05 to detect a minimum change score of 3.8 s. The TUG's minimal clinical important difference (MCID) is between 3.4 and 3.5 s (52, 53). Had the change in TUG scores had been 3.4 s, we would have required a sample of *n* = 17 to detect a clinically meaningful change (alpha = 0.05).

### 4.2. Changes in functional mobility, gait speed, balance, exercise self-efficacy, and health-related quality of life (follow-up vs. baseline)

Changes in outcomes are presented in Table 2. Participants demonstrated statistically significant improvements in functional mobility, balance, self-efficacy for exercise, and health-related quality of life at follow-up compared to baseline measurements. Changes in functional mobility and gait speed each exceeded the minimal clinical important difference _(MCID)_ of the measures (TUG_MCID_ = 3.4–3.5 s, 3-MWT_MCID_ = 0.1–0.2 m/s) (40, 52–54).

**Table 2:** Changes in participants' functional outcome measures between baseline and follow-up.

**Outcomes**	**Baseline, mean (SD)**	**Follow-up (7 weeks) mean (SD)**	**Mean raw score difference (95% CI)**	**Statistical change score difference**
TUG test^a^	17.56 (7.70)	13.32 (4.90)	−4.25 (−6.47, −2.02)	*t*_(14)_ = −4.08, *p* = 0.001
3-MWT^b^	0.83 (0.25)	0.95 (0.34)	0.12 (0.08, 0.16)	*t*_(14)_ = 1.87, *p* = 0.083
Step L leg^c^	7.20 (3.21)	8.93 (3.83)	1.73 (0.50, 2.96)	*t*_(14)_ = 3.03, *p* = 0.009
Step R leg^c^	7.73 (2.31)	9.27 (3.65)	1.53 (0.15, 2.92)	*t*_(14)_ = 2.37, *p* = 0.033
**OEE-2**				
NOEE^d^	2.52 (0.78)	2.13 (0.74)	−0.38 (−0.78, 0.01)	*t*_(14)_ = −2.07, *p* = 0.058
POEEe^e^	4.03 (0.43)	4.22 (0.58)	0.19 (−0.02, 0.0.40)	*t*_(14)_ = 1.96, *p* = 0.071
SEE^f^	7.52 (1.28)	8.50 (0.97)	0.97 (0.33, 1.62)	*t*_(14)_ = 3.27, *p* = 0.006
EQ-5D-5L^g^	0.72 (0.20)	0.83 (0.13)	0.11 (0.02, 0.19)	*t*_(14)_ = 2.73, *p* = 0.016
EQ-5D-VAS^h^	72.33 (18.98)	81.53 (12.14)	9.20 (2.03, 16.37)	*t*_(14)_ = 2.75, *p* = 0.016

CI, Confidence interval.

a TUG measured in seconds; less time indicates better functional mobility.

b 3MWT measured in meters/second; faster time shows better gait speed.

c Number of steps completed in 15 s for the right and left leg; a higher score indicates better balance.

d NOTE, Negative outcome expectancy for exercise, the maximum score possible = 8; a lower score indicates better outcome expectancy.

e POEE, Positive outcome expectancy for training, the top score possible = 5; a higher score indicates better outcome expectancy.

f Self-efficacy for exercise, top score possible = 11; a higher score indicates better self-efficacy.

g Dolan score range 0 = 1; a higher score indicates a better health-related quality of life.

h Visual analog scale 0–100, where 0 is the worst health state and 100 is the best.

### 4.3. Changes in physical activity scores (follow-up vs. baseline)

**Figure 1:**
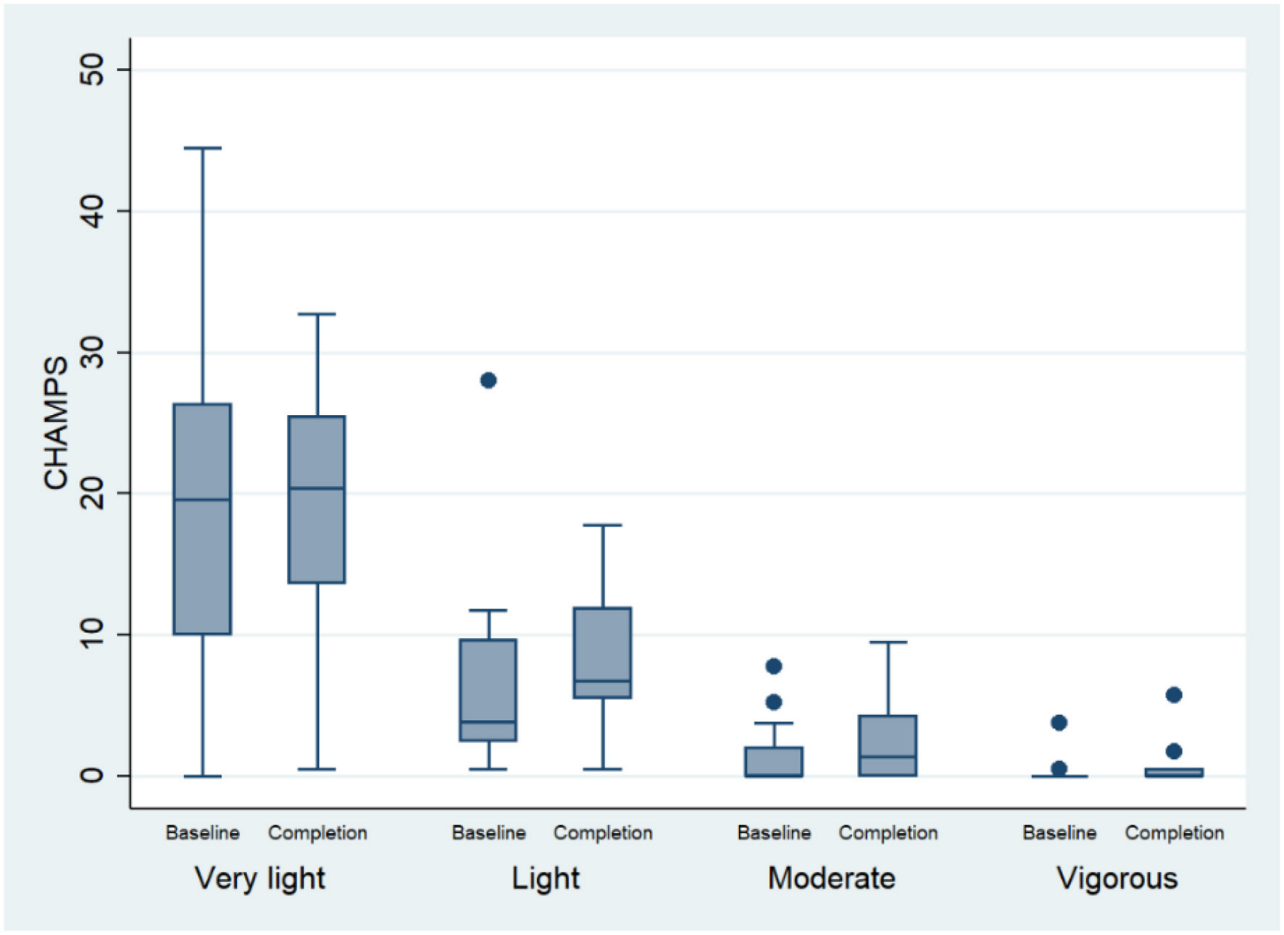
Change in physical activity performance (measured using the CHAMPS) between baseline and follow-up.

Changes in physical activity are presented in Figure 1. After accounting for within-subject correlations averaged over the two data collection points, participants most frequently engaged in very light activities [22.2 h (95% CI, 11.3–33.1)] followed by light [8.4 h (95% CI, 4.2–12.6)], moderate [1.8 h (95% CI, 0.8–2.8)] and vigorous types [0.4 h (95% CI, 0.1–0.8)]. When a change in the total CHAMPS scores was compared over time (follow-up vs. baseline) using the Wilcoxon signed-rank test, a significant increase in activity levels over the DVD intervention was documented (*z* = 2.638; *p* = 0.008). Mixed regression models predicted a 24% increase in overall activity post-intervention [Incidence rate ratio, IRR 1.240 (95% CI, 1.038–1.481); *z* = 2.38, *p* = 0.017]. Further examination of time-activity interactions in the regression model revealed that the overall increase in activity post-intervention was mostly due to the 2.5 times increase in moderate activity [IRR 2.49, (95% CI, 1.42–4.3), *p* = 0.001]. When a participant with a large baseline outlier measure for light activity was removed, light activity levels increased [IRR 1.6 (95% CI, 1.2–2.1), *p* = 0.003]. There were no significant increases in very light or vigorous exercise.

### 4.4. Adherence to the DVD-HEP

**Figure 2:**
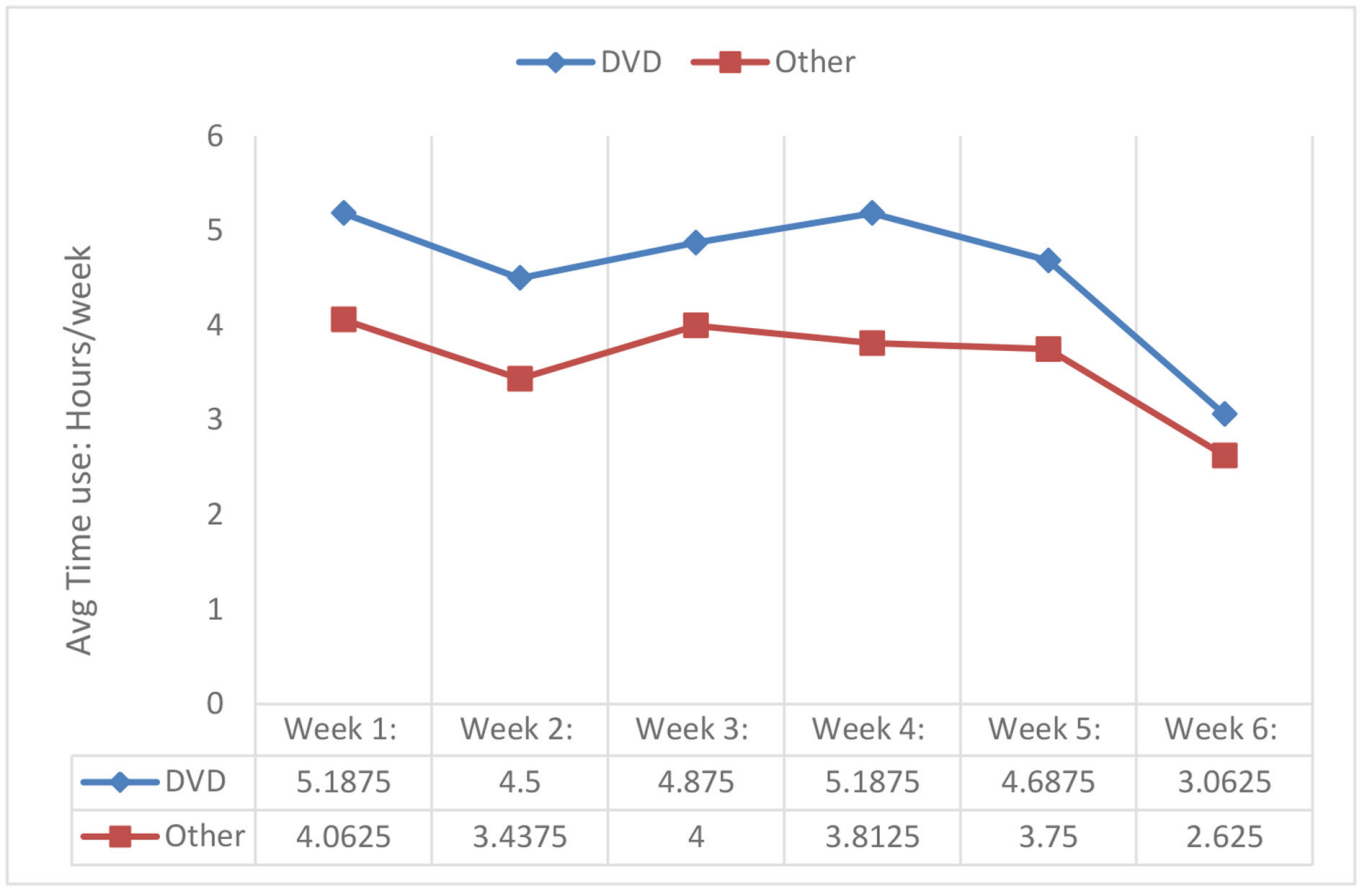
Adherence to the DVD-HEP and other activities (*n* = 14)*. *Data from two participants were missing.

**Figure 3:**
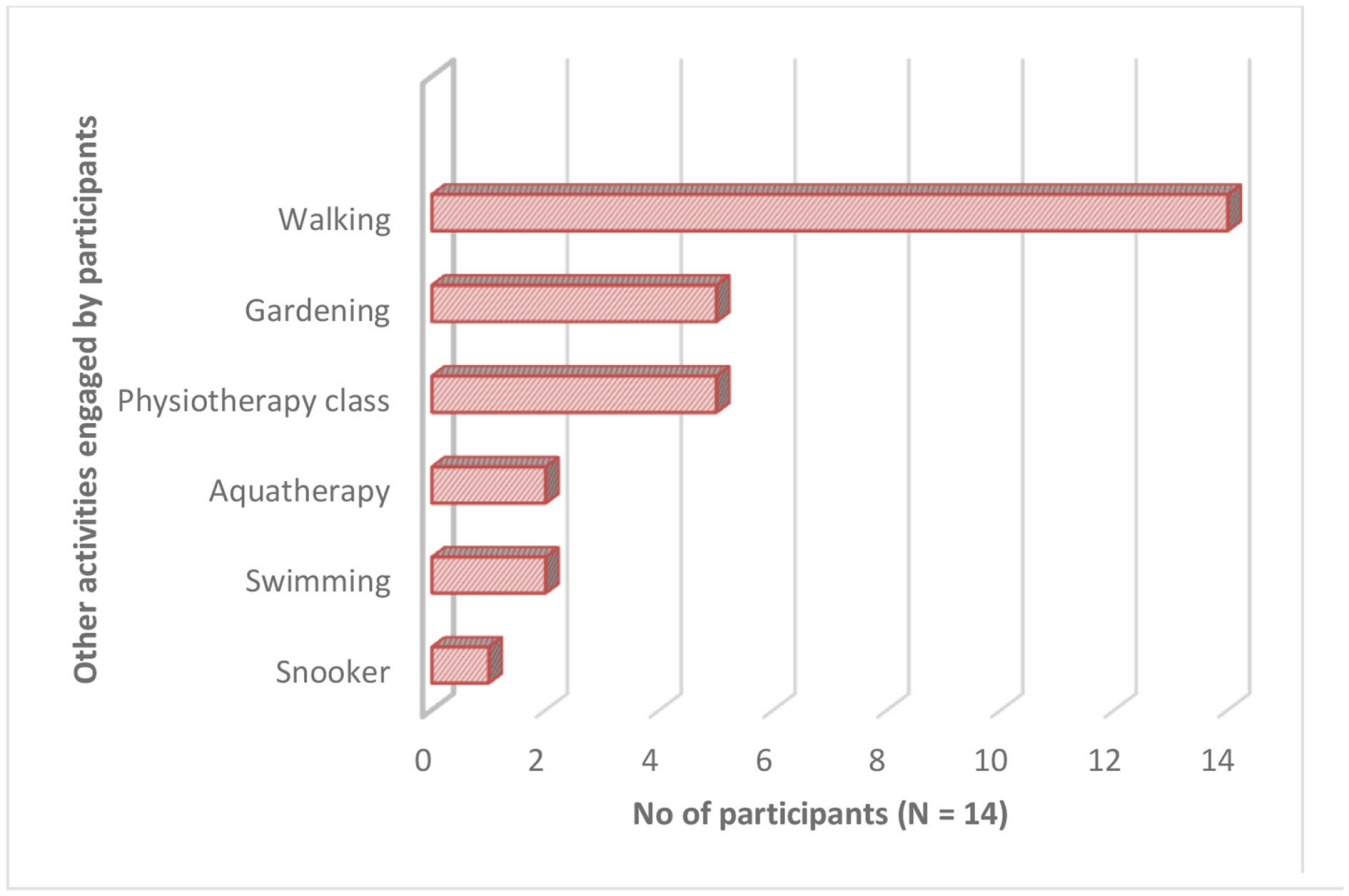
Overview of other activities engaged by participants (*n* = 14)*.

High adherence was confirmed by documentation in participants' exercise diaries, telephone calls, and follow-up visit. As presented in Figure 2, on average, participants reported completing between 3 and 5.1 h of weekly DVD-HEP practice, which was 2–3.5 times the suggested level of exercise frequency prescribed (30-min of the HEP at least three times/week or a total of 1.5 h/week). Further, participants reported performing on average 2.6–4 h of additional weekly physical activity (Figures 2, 3)—with walking being the most frequently reported activity (*n* = 14), followed by attendance at a physiotherapy session (*n* = 5) and gardening (*n* = 5).

### 4.5. Qualitative feedback on the DVD-HEP

A total of 48 phone calls were made to 16 participants (average duration of 25 min) over the course of 6 weeks. The qualitative feedback identified five main themes. The deductive themes were: (i) Enjoyment, self-efficacy, and wellbeing; (ii) Achieving life goals; (iii) Background music as a motivator to adherence; and (iv) Enhanced motor performance and learning: Task goal mastery, multimodal feedback, autonomy to self-regulate learning. The new inductive theme was (v) Preference for in-person support for exercise.

#### 4.5.1. Theme 1: Enjoyment, self-efficacy, and wellbeing

Thirteen participants were strongly positive in describing their DVD-HEP experience and used phrases like, “*Love my DVD, love the music, it makes me feel good... watched it sometimes three times/day and twice on ten days* (P2); “*Very relaxing …. exercising is enjoyable”* (P3 and P4); “*Very good overall”* (P5); “*Very useful”* (P15). Some elaborated further to explain how the DVD-HEP positively impacted their lives. For example, five reported that watching themselves execute the exercise sequels uplifted their mood, “*Viewing the DVD made me feel good”* (P2). Others discussed the boost in confidence and overall sense of wellbeing—which motivated them to continue to practice the techniques as the week progressed (flow and habit formation). Quotes to reflect these sentiments included, “…*The DVD-HEP increased my confidence. I felt so much better after the first week. It was a lack of confidence that made me frightened. Now, I feel good about it; I don't worry even when I walk crooked* (P3)…*Having the DVD motivates me…I wouldn't have bothered otherwise* (P5)*.” Two* others were so impressed with the DVD and print versions of the HEP that they showed them to their family and friends and encouraged them to consult with a physiotherapist to develop a personalized video HEP.

#### 4.5.2. Theme 2: Achieving life goals

Five participants identified that they were achieving more of their own life goals with subthemes of (independence and daily activities) because of improved confidence and physical ability. Sub-themes were overcoming barriers and improved participation. Participants described a sense of achievement in overcoming previously existing barriers, saying, “*Exercises assist with getting up the chair better”* (P9). They also spoke about their improved independence and participation in meaningful daily activities. “*It helps me to go out and about*” (P4)…. “*Yes, it has been much better after doing the exercises. I drive more, didn't notice any pain down (in my) knees”* (P5).

#### 4.5.3. Theme 3: Background music as a motivator to adherence

The *background music* was a source of motivation for some—making exercising at home a “*joyful and fun experience”*—and offered a few participants a “*feel-good factor”* to embed the DVD-HEP into their routine. For example, one participant described her experience as “*…. song by my favorite artist—Love the music with my exercise”* (P1). This participant also said she had “*Advised all older people to try to do their exercises using a video of themselves.”* (P2)

#### 4.5.4. Theme 4: Enhanced motor performance and learning: Task goal mastery, multimodal feedback, autonomy to self-regulate learning

Motor learning was enabled by watching oneself successfully execute each technique (focus on performance technique and task mastery) in conjunction with timely auditory, visual, and proprioceptive feedback provided by the therapist. This included recommendations on correcting techniques or avoiding compensatory movements (voice-over instructions and proprioceptive feedback with the person's own body used as a frame of reference). Thus, multimodal feedback and a sense of task and mastery performance enhanced participants' confidence and motivation to practice the suggested exercise techniques more often—and enabled them to *stretch their learning* as the weeks progressed. Quotes from three participants eloquently articulate this learning experience ”*… Good reminder of how to correctly do the techniques...Toward the end, I watched the DVD more—I learned a little each time... As a result, I see techniques a bit better* (P14)...*Therapist feedback on what to do and why to do it helped me understand how to do the exercise correctly”* (P2).

The relative of a participant with comorbid memory problems reflected on the benefit of the video recording to cue the participant to correctly perform the movement technique saying, “*DVD helps her with exercise—seeing herself do it makes her happy. She loves watching the DVD. She was doing it completely wrong without the DVD due to (sic incorrectly) memory for exercises”* (P4). *Another participant commented that watching the DVD reminded her “....to slow down”* (P6). Further, a participant with chronic hip and knee pain and restricted movements (P12) commented on the benefit of rewinding the DVD a few times to revisit detailed steps involved in some exercise techniques—“*...to ensure that she correctly followed the therapist's recommendations.”* Although this participant commented that the DVD-HEP did not “*cure her long-standing chronic pain problems,”—*she rated the DVD-HEP experience as being “*absolutely brilliant”* (P12).

#### 4.5.5. Theme 5: Preference for in-person support for exercise

Three participants felt that the DVD format did not provide any benefit over the handwritten HEP. The first offered neutral feedback, saying, “*(exercise) was not bad”* (P14), and the second reflected, “*Quite good, don't need to put the DVD every time. Handwritten instructions given by physio were useful reminders”* (P7). The third participant, who had chronic pain and bronchitis, felt in-the-moment monitoring and in-person advice from a physiotherapist would have led to quicker progress, as captured in the quote, “*Enjoyed the exercise initially but not after….need feedback on how to progress rather than do correctly…DVD does not tell me whether I am doing right or not”* (P8).

## 5. Discussion

The study aimed to evaluate the effectiveness of prescribing a tailored video self-modeled DVD-HEP for 6 weeks, on functional mobility, physical activity, exercise self-efficacy, and health-related quality of life, in a sample of frail older adults relative to their baseline scores. Participants demonstrated clinically meaningful improvements in functional mobility and gait speed between baseline and follow-up at seven weeks. The minimal clinical important difference (MCID) of the TUG test is approximately between 3.4 and 3.5 s (52, 53), and the MCID of gait speed (3-MWT) is between 0.1 and 0.2 m/s (54). Clinically meaningful improvements are important as they indicate that the therapy intensity has led to meaningful changes in participants' outcomes (54). Although both these measurements improved from baseline to follow-up, TUG and 3-MWT scores were still below normative values for community-dwelling older adults (40), suggesting our participants' would benefit from continued exercise to improve their ability to function independently in the community. Gait speed is considered such an essential measurement of function that it is referred to as the 6th vital sign (55).

Participants also demonstrated significant improvement in balance, exercise self-efficacy, and health-related quality of life at the 6-week follow-up compared to their baseline scores. Qualitative interviews revealed that the tailored DVD-HEP boosted participants' self-efficacy in successfully performing the exercises and motivated them to practice the advised exercises diligently as the weeks progressed, thus stretching their learning experiences. They reported improved wellbeing and perceived that the DVD-HEP positively impacted their lives. CHAMPS data revealed a 2.5-fold increase in engagement in moderate activities like walking, housework, gardening, and dancing and a 1.3-fold increase in light activity participation over time. Undertaking daily physical activity can augment the gains in a structured exercise program and increase participation in daily activities like shopping or walking, thereby providing positive reinforcement. It is known that exercise after hospitalization can improve functional ability (9, 10). However, older people have low adherence and enjoyment of exercise (12) and report multiple problems in recovery after hospital discharge, including engaging in exercise (13). The functional decline after hospitalization is a significant problem for older people, with multiple studies identifying that older people find it challenging to adapt to daily life after discharge due to difficulties in performing their daily activities and that even after 12 months, they continue to experience a functional decline (5, 56, 57). Programs for older people after hospital discharge that focus on providing support and rehabilitation have been identified as an urgent priority (58, 59). Innovative programs that encourage self-directed learning are needed.

Each DVD-HEP was informed by an initial face-to-face assessment by an experienced physiotherapist and was based on validated performance measures, participant goals, and functional needs. As part of the tailoring process, we included a personalized introduction to the HEP, evidence-based exercise components to address individual goals, visual cues (gestures), and specific instructions that each participant should look out for (contraindications and compensatory movements) while practicing the HEP. This was designed to simulate the experience of a face-to-face physiotherapy session.

The COVID-19 pandemic has highlighted the value of innovative strategies, including telehealth exercise programs, for preventing functional decline among older people during periods of social distancing and quarantine (60). Emerging evidence suggests that digital health modalities such as telephone calls or videoconferencing, which depend on synchronous contact with a health professional (20) and voice-controlled intelligent personal assistants (VIPAs, using Amazon Alexa), are feasible in older people. However, several barriers to adherence to the latter have been reported (namely, poor internet connectivity, voice recognition inaccuracy, and privacy issues) (61). Importantly, these modalities do not provide personalized exercise prescriptions with hands-on feedback as in our novel, tailored, self-modeled video HEP. Video self-modeling might be a novel way to improve the telehealth model by arranging an initial face-to-face “hands-on” session with the physiotherapist.

Given the amount of time and resources devoted to the creation of a tailored DVD for each participant and the suggestion that these may need to be adjusted as the individual's physical fitness changes, it is essential to acknowledge that a self-modeled audio-visual approach comes with additional demands on clinician time and workload. Technological advances, including software options for collating videos, have significantly developed. Almost all modern computers, tablets, and even smartphones come preloaded with advanced video editing software. It is also relatively easy to upload videos online to YouTube and other platforms or embed them into streaming platforms equipped with robust governance structures. For the 27–49% of Australians aged 75 and over, who do not access the Internet (62), making a high-quality video and converting it to portable hardware is quick, economical, feasible, and requires little technical expertise. The current study's findings call for further investigation into the adherence and maintenance of longer, tailored, self-modeled video HEP and how therapists could use such modalities more routinely in clinical practice, like the possibility of a video-delivery HEP as a substitute for some face-to-face treatment sessions. Future studies comparing the impact, sustainability, and cost-effectiveness of different modalities of self-modeled video-based digital HEP are desirable to ensure that all older people have access to safe, efficient, and high-quality care aligned to their needs, preferences, and learning styles.

We can draw on several learning theories, including the cognitive theory of learning (63), and the Optimizing Performance Through Intrinsic Motivation and Attention for Learning (OPTIMAL) theory of motor learning (64), to explain the current study's findings. Cognitive theorists would argue that it was likely that our tailored self-modeled DVD-HEP was designed to be situated within each individual's “*zone of proximal development”* (63). Accordingly, each participant was motivated to self-critique, create personal learning points while watching the video, and engage in more self-regulated learning experiences (65). According to the OPTIMAL theory of motor performance, the DVD-HEP facilitated motor performance and learning by encouraging practice conditions that promoted enhanced expectancies, autonomy, and external focus of attention (66) and motivated participants to practice and adhere to the physiotherapist's advice, thereby facilitating the consolidation of motor memories (67). Applied to the current study, watching oneself successfully execute exercises on the video footage could have encouraged participants to focus their attention on task goal mastery, thereby enhancing their expectations of performance and challenging their negative exercise expectations grounded in fear and perceptions of task difficulty (30, 68). Consequently, participants' negative outcome expectancy of exercise scores—objectively measured using the OEE-2 scale (in terms of avoiding exercise because of shortness of breath, pain, fear of falling or getting hurt, and stress on the heart) reduced after the 6-week DVD-HEP intervention.

The DVD-HEP was designed to allow participants to choose their exercise-free days and the frequency of repetitions and sets, which could have given participants a sense of agency or control (69) and encouraged self-determination of when, how often, and how intensely they exercised (70). Such practice conditions could have influenced participants' motivation to practice (71) and enhanced their self-efficacy in successfully executing the exercise routine shown in the video (72). Also, the inclusion of specific and targeted audio-visual and proprioceptive instructions and encouragement by the physiotherapist in the video recording alongside participant-selected background music was reported by participants to transform the DVD-HEP exercise routine into a “*joyful and fun experience”* and offered a few participants a “*feel-good factor.”* This, in turn, motivated most participants to engage with the DVD-HEP routinely—thus suggesting why there was high adherence to the intervention in our current sample. The current trial adds to the growing evidence base on the potential efficacy of self-modeling videos of task mastery on improved motor performance in frail older adults in the community (64).

Given that our sample improved relative to their baseline scores over a relatively short 6-week period, a follow-up visit to modify the exercises and re-video might be necessary for sustained programs. While the DVD-HEP was considered favorably by most, three of the 16 participants felt the medium did not allow for in-the-moment in-person monitoring of progress that an in-person physiotherapy session would allow. This suggests that self-modeled video-HEP may not suit all older adults. Our findings validate those of a recent observation study (23) and highlight the benefits of consultation with individual patients to explore their preferences and collaboratively design tailored programs aligned to each individual's needs, preferences, and interests and deliver them using mediums that are congruent to the person's learning preference, style, and digital literacy (17, 73).

## 6. Strengths and limitations

All participants were made aware of the health benefits of including exercise in their daily routine and were provided personalized guidance on avoiding and managing potential risk situations while exercising (e.g., fatigue, postural hypotension). However, at the time of enrolment, all participants were not engaged in any exercise or physical activity program at home or in the community. Changes in primary and secondary outcomes were measurable and clinically significant, therefore, may be a result of participation in the DVD-HEP intervention and changes in participants' lifestyles during the intervention, but the chance of natural improvement cannot be eliminated.

Some limitations of this study must be acknowledged when interpreting the findings. We originally planned to enroll more participants; however, the trial coincided with a change in patient flow through services at the participating hospital due to the opening a new hospital. Further attempts to scale up the DVD-HEP model could be considered. This could involve following up with participants after 6-weeks, enrolling more participants in an outpatient setting, or incorporating video prescriptions as part of the usual care. We used a single group and a sample of convenience. The design and sample size were initially designed to evaluate the intervention with a control group. However, we did not have a non-intervention control group to assess time-dependent changes and explore the contribution of factors on outcomes (e.g., sex, comorbidity, level of frailty). Also, DVD-HEP adherence was self-reported *via* a daily diary, which may be susceptible to overestimation bias. In addition, we did not objectively measure exercise fidelity. Future studies could use objective measurements of adherence, such as self-monitoring activity devices or videoconference monitoring of exercise, and use dynamometers to measure changes in physical function. Future studies could explore the influence of several confounders—such as the level of comorbidity or frailty and how sociodemographic variables such as education levels and sex influenced adherence and outcomes. A genuine rapport was built between the treating physiotherapist and our study's participants, encouraging them to feel safe sharing their views (50). While it could be considered a limitation not to have a separate interview, it was considered that the physiotherapist was their trusted clinician, and participants would feel comfortable responding over the phone. This may have been a limitation as some may have felt reluctant to respond negatively to the therapist (74). We acknowledge potential assessor bias as post-test assessments were completed by the same physiotherapist who did the baseline assessment and follow-up telephone calls. We tried to lessen assessor bias by using standardized, validated objective outcome measurements sensitive to change.

## 7. Conclusion

Older adults who were prescribed a tailored self-modeled DVD-HEP (which involved one face-to-face session with a physiotherapist and three follow-up phone calls) demonstrated functional improvement compared to their baseline assessment after 6 weeks of completing the program independently at home. Their adherence to the exercise program exceeded the recommended levels—suggesting participants were intrinsically motivated to use the DVD-HEP. Given advances in digital technology, future comparative studies on the efficacy of tailored self-modeled video HEP to other formats are needed to serve older adults optimally. Future healthcare systems will expect older adults to assume a more dominant role in their health and rehabilitation care. With appropriate professional guidance, based on the current study's findings, tailored self-modeled digital HEP could provide a new and novel avenue to evaluate how to provide sustainable, high-quality health professional input alongside independent exercise. This study suggests that tailored self-modeled digital HEPs may stimulate self-management and facilitate motivation, a sense of responsibility, and confidence to practice. This, in turn, can optimize functional outcomes and improve long-term health behaviors in older adults.

## Data availability statement

The raw data supporting the conclusions of this article will be made available by the authors, without undue reservation.

## Ethics statement

Ethics clearance for the study was obtained from the University of Notre Dame Human Research Ethics Committee (HREC) (Reference Number: 015146F) and the South Metropolitan Health Service (SMHS) HREC (Reference Number: 15-190). All participants provided written informed consent to be included in the study.

## Author contributions

A-MH, JC, and DB conceived and designed the study. DB led site procedures. A-MH led the data collection with assistance from J-AH. A-MH, J-AH, JC, DB, KS, and SV contributed to the data collection, analysis, and interpretation. SV, A-MH, and J-AH drafted the manuscript with assistance from JC. All authors provided feedback on the manuscript drafts, read, and approved the final manuscript submitted.
