# Supplementary material for: Prescribing tailored home exercise program to older adults in the community using a tailored self-modeled video: A pre-post study
Source: Front Public Health. 2022 Dec 22;10:974512. doi: 10.3389/fpubh.2022.974512 (PMC9815032; doi:10.3389/fpubh.2022.974512)
Supplement: Supplementary file 1 [file Data_Sheet_1.docx]

Supplementary File 1: Fidelity and feasibility considerations in designing the DVD intervention

| Intervention design | Technique used | Consideration |
| --- | --- | --- |
| Exercises | Individually tailored for each participant | An individual exercise program from a therapist. It was personally tailored to meet the individual's goals, preferences, therapeutic needs, and baseline assessment findings |
| Exercise practice | Participant practiced exercises | Participants practiced exercise techniques first and then corrected with the therapist before being recorded |
| Exercises | 8-10 exercises for each participant, warm-up, main exercises, and cool down | Exercises follow evidence-based guidelines for completing an exercise program |
| Environment | Use of chairs, walls, simple equipment (sturdy surface, portable resistance bands | Replicate equipment that would be readily available in a home environment |
| The physical therapist in the video | Shown gesturing, with hands-on key body points as necessary, to facilitate correct movement | A therapist uses clinical judgment about how best to guide the correct movement |
| Verbal cueing | Voice over cues and information to help identify the correct technique in the video | Ensure that the patient can both see the correct technique on the video and hear it also as it is being shown on the DVD |
| Length of time to video | The whole appointment is completed in approximately one h (30-min preceding enrollment and administration) | Complete DVD filming in what would be a typical first clinical appointment and also not to fatigue participant |
| Video procedure | Physiotherapist researcher filmed with student assistant; tripod set up for filming | Completed in the outpatient department needed to be filmed with other areas in use e loudness and space considerations |
| Visual feedback | Complete footage of three repetitions of the complete exercise from start to finish | Shown from a close angle to relevant exercise and then from a whole-body shot |
| Music | Chosen by the participant, then entered into the format after the video footage was cut | To facilitate enjoyment and retain the individual nature of DVD. Participants were asked to provide a motivational instrumental music track to be played in the background. The volume of the background music was adjusted to ensure that it did not overpower or clash with the verbal cues. In addition, instrumental music was selected to minimize participant distraction or unintended attention to voices and sung lyrics. |
| DVD format | Title and introduction in stills, section headings with exercise numbers burnt onto DVD format | Large print used plain black on white background, slow change of still instructions, time to read |
| Effective use of time | Film rendered onto DVD after participant left | The patient did not need to be there after the recording had been completed |
| Video rendering | Footage edited with voice-over corrections and applied to a standard DVD format in iMovie (iMovie '11 [version 9.0] for Mac, 2010 Apple Inc., Cupertino, California). DVD rendering techniques for both visual and audio data | Ensure the highest possible quality is available on the DVD format to ensure participant enjoyment, able to be viewed on a standard TV/DVD player |

Adapted from Moran and colleagues(30)

Supplementary File 2: Semi-structured phone call interview template

Phone Call 1: Weeks 2, 4 and 6

- Physiotherapist to check if the participant has received the DVD in the mail and used the DVD (Week 2 only)
- Problem solve technical issues or clinical issues expressed by participant
- Probe the impact on life goals, everyday activities, and confidence in exercising

What has worked well in the DVD program? (Probes: personalization, motivation, visual feedback, reference point, ease to follow, background music)

__________________________________________________________________________________________________________________________________________________________________________________________________________________________________

What is not working well in the DVD program? (Probe: technical issues and clinical issues)

__________________________________________________________________________________________________________________________________________________________________________________________________________________________________

What are your thoughts at the moment about the DVD program?

__________________________________________________________________________________________________________________________________________________________________________________________________________________________________

Has the DVD program helped you to exercise?

__________________________________________________________________________________________________________________________________________________________________________________________________________________________________
